# Potential Diagnostic and Research Applications of a Recombinant Antibody Directed Against Ferrated Triacetylfusarinine C from *Aspergillus fumigatus*

**DOI:** 10.3390/jof12050342

**Published:** 2026-05-06

**Authors:** Marie Dwyer, Rebecca A. Owens, Claudia Garcia Revuelto, Kieran G. Walshe, Cathal M. Murphy, Nicola M. Moloney, Sean Doyle

**Affiliations:** 1 Department of Biology, Maynooth University, W23 XY3X Maynooth, Ireland; mg.dwyer.12@gmail.com (M.D.); rebecca.owens@mu.ie (R.A.O.);; 2 Accuplex Diagnostics Limited, A85 CD51 Ratoath, Ireland

**Keywords:** siderophore, recombinant antibody, ELISA, *Aspergillus* diagnostics, immunotherapy, invasive aspergillosis

## Abstract

Although *Aspergillus fumigatus* has been identified as a critical fungal pathogen by the World Health Organization, diagnosis of the various types of aspergilloses remains unsatisfactory. New biomarkers of disease and accessible test systems are needed. Moreover, new treatment concepts are required to address infections caused by this invasive pathogen. Siderophore production by *A. fumigatus* offers opportunities for both improved diagnosis and potential therapy. Here, we report the development of a competitive ELISA that detects ferrated triacetylfusarinine C (FeTAFC) using a recombinant monoclonal IgG [anti-FeTAFC]. The FeTAFC ELISA can detect FeTAFC in human urine, a matrix proposed to be especially suitable for disease diagnosis because of the non-invasive nature of specimen collection. In addition, a novel assay was developed to assess the in vitro inhibitory properties of the IgG [anti-FeTAFC] towards *A. fumigatus* mutant and wild-type growth under iron-limiting conditions. The growth of *A. fumigatus* Δ*sidD*, deficient in TAFC and precursor fusarinine C (FsC) biosynthesis, was significantly reduced (*p* = 0.0003) in the presence of the antibody. While the growth of *A. fumigatus* wild-type was less affected in the presence of the antibody, the ratio of secreted TAFC:FsC was increased due to increased conversion of FsC to TAFC—likely because of extracellular complexation of FeTAFC by the IgG [anti-FeTAFC]. This work shows the utility of the IgG [anti-FeTAFC] for the detection of *A. fumigatus* and initial evidence for limiting fungal growth by attenuation of FeTAFC uptake.

## 1. Introduction

*Aspergillus fumigatus* is an opportunistic fungal pathogen responsible for several devastating pulmonary diseases. The most severe of these diseases, invasive pulmonary aspergillosis (IPA), has a mortality rate of between 30 and 90% depending on the underlying condition and early and appropriate treatment, including access to triazoles or amphotericin B [1]. Unspecific diagnostic criteria often lead to a delay in treatment [2], and the treatment options available are limited to the use of azoles and amphotericin B [3]. These treatments are known to often have toxic side effects [4].

The European Organisation for Research and Treatment of Cancer and Mycoses Study Group Education and Research Consortium (EORTC/MSG) updated the diagnostic criteria for invasive aspergillosis (IA) in 2019 [2]. These include clinical features and mycological evidence that indicate potential IA. Clinically, a lung computerised tomography (CT) scan will likely show dense lesions, consolidation, cavities, and air crescent sign as crescent-shaped cavities filled with air. Mycological evidence includes recovering fungal elements from sputum, bronchoalveolar lavage (BAL), and bronchial brush or aspirate. These can be detected microscopically or recovered by culturing of the samples. Invasive pulmonary aspergillosis (IPA) can also be diagnosed using the galactomannan (GM) antigen assay or PCR, although PCR requires at least two positive tests to qualify a patient as positive for IPA. However, these criteria have limitations. Firstly, they were developed based on diagnosis in solid organ transplant, stem cell, and cancer patients. As patients with suspected fungal infections in the ICU have a variety of underlying conditions, these criteria are not always applicable [5]. Only two of the criteria listed are specific to IPA, as fungal hyphae are difficult to distinguish, and the features listed for CT scans are also diagnostic criteria for other mould infections. Metagenomic next-generation sequencing (mNGS) has also been suggested as a diagnostic method for IPA [6]. The two specific assays, PCR and GM antigen assay, also have limitations. Both assays can yield false positives, and PCR is known to have low specificity [7]. The GM antigen assay shows high sensitivity and specificity on cerebral spinal fluid [8] and BAL samples [9], but shows less sensitivity with serum samples [10] and appears to be less sensitive if patients had been given prophylactic antifungal treatment [11]. *Aspergillus* PCR is shown have higher specificity when carried out on BAL samples [12]. BAL, which is the sample used for most IPA diagnostic testing, is perceived to be somewhat invasive for clinical patients and may cause lung damage [12]. Serum-based assays generate results which appear to be less specific, and none of the listed criteria for IPA diagnosis are carried out on urine samples. Conversely, urine testing is a suitable alternative as the collection of urine from a patient is a non-invasive process [13]. However, it is important that any assay which proposes to use urine as a matrix is first subjected to spike sample urine analysis, as factors such as the pH and ion content of urine can interfere with analysis [14]. Given the limitations of the current IPA diagnostic assays, the development of new non-invasive assays, with high levels of sensitivity and specificity, is essential.

It has been proposed that small molecules secreted from *A. fumigatus* during infection could be used as biomarkers of IPA, such as the epipolythiodioxopiperazine gliotoxin [15,16] and its methylated analogue bismethylgliotoxin [17,18]. Herein, we describe the development of a diagnostic assay based on another prospective small molecule urinary biomarker triacetylfusarinine C (TAFC), an *A. fumigatus* siderophore [19,20,21,22,23]. TAFC is a secreted, ferric iron-chelating siderophore that has been shown to be essential for the virulence of *A. fumigatus* [24] and is present during *A. fumigatus* infection as a result [25]. In fact, several studies have revealed that in murine IPA models, TAFC can be detected in urine using mass spectrometry (MS) [19,21]. Similarly, MS analysis of the urine of IPA patients showed that a TAFC/creatinine index is useful for the diagnosis of IPA [20,23]. MS is a slow, labour-intensive process requiring specialist equipment that is not yet available in most hospital settings. Therefore, the availability of a more rapid and accessible assay, such as ELISA, would be ideal. To that end, a recombinant monoclonal IgG [anti-FeTAFC] was generated [26] with the potential for TAFC or ferrated TAFC (FeTAFC) immunodetection; however, this assay has not yet undergone validation. Such validation has now been completed and is presented herein.

Relatedly, several strategies involving the use of monoclonal antibodies (MAbs) to inhibit the growth of fungi exist and mainly involve the use of MAbs against fungal cell wall components. MAb C7 was developed against the cell wall mannoprotein in *Candida albicans.* This MAb was shown to inhibit adhesion and germination [27]. AK-14, a MAb against *A. fumigatus* glycoprotein, was shown to reduce the adhesion of hyphae to polystyrene [28], which could help prevent the adhesion of *A. fumigatus* hyphae to medical devices. MAb 1D2 against an *A. fumigatus* cell wall glycoprotein was shown to inhibit the growth, attachment, and germination of *A. fumigatus* conidia and caused damage to hyphae [29]. A MAb against *A. fumigatus* cell wall glycoprotein, MAb A9, was shown to reduce hyphal development and the duration of spore germination [27]. Herein, we also explore the potential growth-inhibiting properties of the recombinant anti-siderophore monoclonal antibody [26] using novel assay formats.

## 2. Materials and Methods

### 2.1. Coating of ELISA Plates with Ferrated Diacetylfusarinine C-Bovine Serum Albumin Conjugate (FeDAFC-BSA)

All chemicals were from Sigma-Aldrich, St. Louis, MO, USA unless otherwise stated. Ferrated diacetylfusarinine C-bovine serum albumin conjugate (FeDAFC-BSA) was prepared as described previously [26]. Microtitre plates were coated with 100 μL/well FeDAFC-BSA (0.12 μg/mL in 50 mM sodium carbonate buffer pH 9.6) before overnight incubation at 4 °C, after which they were washed with PBS–Tween 20 and blocked using 200 µL/well blocking solution for 1.5 h at 37 °C. The blocking solution was removed, and the microplates were dried at 37 °C overnight and stored sealed at 4 °C prior to use.

### 2.2. Conjugation of Recombinant Monoclonal IgG [Anti-FeTAFC] to Horseradish Peroxidase (HRP)

Conjugation of HRP to recombinant monoclonal IgG [anti-FeTAFC] [26] first required the addition of thiol and maleimide groups to the recombinant monoclonal IgG [anti-FeTAFC] (hereafter IgG [anti-FeTAFC]) and HRP, respectively. A total of 3.25 µmol N-succinimidyl S-acetylthioacetate (SATA) (3.1 µL; 242.7 mg/mL) in dimethylformamide (DMF) was added to 400 nmol HRP (4 mL; 4 mg/mL) in PBS-EDTA pH 7.8 and reacted at 20 °C for 1 h. Following the reaction, the activated HRP was dialysed against PBS-1 mM EDTA pH 6.8. The IgG [anti-FeTAFC] (1 mL; 6.77 mg/mL) was dialysed against PBS-1 mM EDTA pH 7.8 and diluted to a final concentration of 1 mg/mL. A total of 472 nmol succinimidyl 4-(N-maleimidomethyl)cyclohexane-1-carboxylate (SMCC) (18.8 µL; 8.4 mg/mL) in DMF was added to 16.7 nmol IgG [anti-FeTAFC] (2.5 mL; 1 mg/mL) and incubated at 20 °C for 40 min. The activated antibody was then dialysed against PBS-1 mM EDTA pH 6.8. Thiol groups on HRP were exposed by 0.5 M hydroxylamine (100 µL) addition to SATA-modified HRP (1 mL; 3.5 mg/mL), followed by 1 h in the dark at room temperature. SMCC-modified IgG [anti-FeTAFC] (2 mL; 2 mg/mL) was then added, allowed to react for 6 h at room temperature in the dark, and the reaction terminated as described in [30]. The antibody conjugate was then dialysed against PBS, and a master stock was produced by a 1:1 dilution in glycerol and stored at −20 °C. This stock was diluted 1/1000 further in StabilZyme antibody stabilising solution (Surmodics IVD Inc., Eden Prairie, MN, USA). This intermediate stock was stored at 4 °C until testing by immunoblot against FeDAFC-BSA (0–200 ng).

### 2.3. ELISA Procedures

A two-step ELISA procedure was carried out as described [26]. A one-step ELISA procedure was developed as follows. The FeTAFC and antibody conjugate were diluted to appropriate concentrations in assay diluent. FeTAFC standards (0–1000 ng/mL) and test specimens were added to the plates first, followed by the antibody conjugate (1/20,000–1/40,000) to optimise antibody conjugate dilution. After 1 h incubation at 37 °C, FeTAFC ELISA plates were washed and substrate tetramethylbenzidine (TMB) (Moss Inc., Franklin Park, IL, USA) was added for 10 min, followed by ELISA Stop Solution (1N H_2_SO_4_). The absorbance of the solution in each well was measured at 450/630 nm. Assays here forward use a 1/30,000 IgG [anti-FeTAFC]-HRP dilution, unless otherwise stated. GraphPad Prism Version 9.1.2. was used for the construction of all graphs and statistical analysis.

### 2.4. Evaluating the Intra-Assay and Inter-Assay Precision and Human Urine Compatibility of the One-Step ELISA

The antibody conjugate (110 µL) was pre-incubated (1 h, 37 °C) with 6 specimens, each of 1000, 250, and 63 ng/mL FeTAFC (110 µL). The combined samples were then added to a single plate in duplicate (200 µL /well), and the one-step ELISA was carried out. The %CV (% coefficient of variation) of the test specimens was calculated as follows: %CV = (standard deviation/mean) × 100%. For inter-assay precision, the antibody conjugate (110 µL) was incubated with 1000, 250, and 63 ng/mL FeTAFC (110 µL). The specimens were then added to the plate in duplicate (200 µL /well), and the ELISA was carried out. This procedure was replicated over eight different assays and the %CV of the test specimens was calculated. Urine samples were obtained from 12 volunteer donors and stored at −20 °C before use. Ethical permission was obtained from the Maynooth University Biomedical and Life Sciences Research Ethics Committee (reference number: BSRESC-2021-2425581) for the collection and use of human urine. One of the urine samples was selected randomly and a serial dilution (0–1000 ng/mL) of FeTAFC was carried out in the sample and in the assay diluent. FeTAFC was also diluted to 1000, 250, and 50 ng/mL in each of the urine samples and in the assay diluent. These samples and titrations were assayed using the one-step ELISA procedure.

### 2.5. Selectivity of the FeTAFC ELISA

Serial dilutions (0–1000 ng/mL) of ferrated ferricrocin (FeFC) [31], FC, ferrated fusarinine C (FeFsC), FsC, and FeTAFC were prepared in assay diluent. These samples were then analysed using the two-step ELISA procedure. Separately, a serial dilution (0–1000 ng/mL) of FeTAFC, TAFC, and gallium–TAFC (GaTAFC) were prepared in assay diluent. A two-step ELISA comparison of the TAFC to the FeTAFC titrations, followed by a one-step ELISA comparison of the TAFC and GaTAFC to the FeTAFC, were carried out.

### 2.6. Media for the Growth of A. fumigatus Under Iron-Deplete Conditions

*A. fumigatus* Δ*sidD* was kindly gifted by Professor Hubertus Haas (Innsbruck, Austria). The glassware used in the preparation of media for the growth of *A. fumigatus* (wild-type ATCC46645 (ATCC, VA, USA) or Δ*sidD*, deficient in FsC and TAFC biosynthesis [32]) was incubated with 1.5 mM ethylenediaminetetraacetic acid (EDTA) for 3 h, followed by overnight incubation with 5% (*v*/*v*) HCl to remove iron. All media and components were produced using deionised water. A total of 1 L of iron-deplete media was made using Formedium Yeast Nitrogen Base without amino acids and without iron (Formedium Ltd., Norfolk, UK) (1.9 g), supplemented with glucose (10 g) and adjusted to pH 6.5 prior to sterilisation by autoclave at 115 °C for 30 min. For solid culturing, agar (12 g) was added prior to autoclaving. The solution was then further supplemented with sterile 0.3 M L-glutamine (66.3 mL) and 118 µg/mL bathophenanthrolinedisulfonate (BPS) (200 µM final concentration). Statistical analysis used for all growth assays was two-way ANOVA.

### 2.7. The Effect of the IgG [Anti-FeTAFC] on the Growth of A. fumigatus ΔsidD

Iron-deplete agar (6 mL/well) was added to 6-well culture plates. In total, 100 μL of *A. fumigatus* Δ*sidD* conidia (500 conidia/mL) was spread over each well and allowed to dry. A 10 mm sterile filter disc (Whatman, Kent, UK) was added to the centre of each well and sterile FeTAFC diluted to 100, 50, 20, 10, and 5 pmol/disc was added to the discs in duplicate, based on preliminary optimisation experiments and data (Supplementary Figures S1 and S2). The plates were incubated at 37 °C for 48 h to determine the amount of FeTAFC required to enable growth. It was observed that 50 pmol/disc FeTAFC was needed to restore the growth of *A. fumigatus* Δ*sidD*. Following this, an agar assay with supplementation of FeTAFC at 50 pmol/disc and either PBS, IgG [anti-FeTAFC], or BSA at 100 and 200 pmol/disc was established in the same manner as before. The plates were incubated at 37 °C for 48 h. Blue food dye (Goodall’s, Dublin, Ireland) was used as a contrast stain to allow the mycelia to be imaged, and the growth was measured using imageJ (FIJI) version 2.1.0. analysis.

### 2.8. The Effect of IgG [Anti-FeTAFC] on the Growth of A. fumigatus Wild-Type

Fluorescent FeDAFC was synthesised to confirm the siderophore uptake capacity of *A. fumigatus* (Supplementary Data). Subsequently, assays were performed to establish the minimum FeTAFC concentration needed for growth restoration of *A. fumigatus* wild-type. Following this, agar assays with supplementation of FeTAFC at 20 pmol/disc and either PBS, IgG [anti-FeTAFC], or BSA at 40 and 100 pmol/disc was set up in the same manner as described in Section 2.7. Plates were incubated at 37 °C for 48 h. Blue food dye was used as a contrast stain to allow the mycelia to be imaged, and the growth was measured using imageJ analysis.

### 2.9. The Effect of IgG [Anti-FeTAFC] on the Growth of A. fumigatus in Microwell Assays

A total of 44 μM IgG [anti-FeTAFC] and BSA (45.5 μL) were diluted to 1 μM in iron-deplete media. Serial dilutions (1–0 μM) were carried out on the protein samples in iron-deplete liquid media. *A. fumigatus* conidia were added to each of the solutions to a final concentration of 2.5 × 10^5^ conidia/mL, and 200 μL of the solutions were added to wells of a 96-well flat bottomed microwell plate in duplicate. The plates were incubated static for 24 h at 37 °C and imaged under an Olympus SZX16 microscope with an Olympus SDF PLAPO 2XPFC camera attachment (Olympus, Tokyo, Japan).

### 2.10. The Effect of IgG [Anti-FeTAFC] on the Growth of A. fumigatus in 10 mL Cultures

PBS, IgG [anti-FeTAFC], or BSA were added to iron-deplete media at a final concentration of 45 μM. The solutions were inoculated with *A. fumigatus* conidia to a final concentration of 2.5 × 10^5^ conidia/mL. In total, 10 mL of the culture was added to each of 5 × 50 mL centrifuge tubes. The tubes were incubated at 37 °C for 48 h with shaking. The mycelia were collected and the weight recorded. The supernatant was collected and filtered through 50 kDa spin filters to remove IgG [anti-FeTAFC] or BSA. FeSO_4_ was added to the samples at 1.5 mM and allowed to incubate at room temperature for 40 min. Supernatants were analysed by UV–vis spectroscopy and RP-HPLC (Agilent, CA, USA) at 440 nm, as previously described [33].

## 3. Results

### 3.1. Antibody Conjugate Synthesis, Functionality and Optimisation in a One-Step FeTAFC ELISA

**Figure 1:**
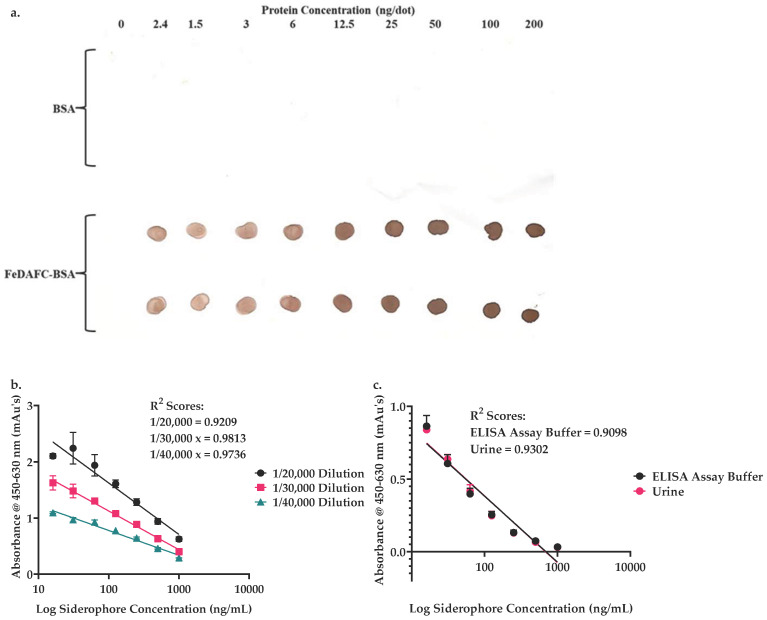
IgG [anti-FeTAFC]-HRP direct conjugate functionality. (**a**) IgG [anti-FeTAFC]-HRP direct conjugate reactivity with immobilised FeDAFC-BSA and not the BSA carrier protein, as assessed using immunoblot analysis. FeDAFC-BSA was titred from 0 to 200 ng/dot. At all concentrations of FeDAFC-BSA, the signal is seen following the addition of DAB substrate, but no signal is observed for the immobilised BSA. This confirms direct conjugate functionality and FeDAFC specificity. (**b**) The mean absorbances of FeTAFC titration with IgG [anti-FeTAFC]-HRP conjugate 1/20,000, 1/30,000, and 1/40,000 dilutions were used to construct standard curves. The x-axis was displayed on a log scale, and a semi-log line of best fit was used. The R^2^ scores for each were used to determine that the optimum IgG [anti-FeTAFC]-HRP dilution as 1/30,000. (**c**) Assessment of the viability of urine as a matrix for the FeTAFC ELISA, four replicates of an FeTAFC titration in either urine or ELISA assay buffer were analysed. The mean absorbance at each concentration was used to make a standard curve of both the samples in urine and in ELISA assay buffer. The x-axis was displayed on the logarithmic scale, and a semi-logarithmic line of best fit was used. Overlap of the standard curve indicates there is no negative matrix effect with urine as a matrix.

After SATA and SMCC activation, Aldrithiol-4 thiol assay [30] showed that the ratio of SH/HRP was 1:1.05 and the ratio of maleimide to IgG [anti-FeTAFC] was 1:5.4, enabling stable thioether antibody conjugate (IgG [anti-FeTAFC]-HRP) formation. Immunoblot analysis confirmed antibody conjugate synthesis and the ability to detect FeTAFC at the lowest concentration tested, 1.5 ng/dot, but there was no detection of BSA (Figure 1a). This confirmed successful conjugate synthesis and functionality, as well as the unprecedented conjugation of IgG [anti-FeTAFC] directly to an enzyme label. In the one-step ELISA, an antibody conjugate dilution of 1/30,000 yielded the best semi-log fit (R^2^ = 0.9813) (Figure 1b), and so this dilution factor was chosen for future analyses.

### 3.2. High Intra- and Inter-Assay Precision Was Observed at FeTAFC Concentrations Above 250 ng/mL

The results of the intra-assay precision assay are seen in Table 1. At the low FeTAFC concentration (63 ng/mL), a %CV of 42% was observed. However, at the medium concentration (250 ng/mL) and high concentration (1000 ng/mL), the %CVs were 25% and 13%, respectively. This shows there was acceptable intra-assay precision at these concentrations, albeit with 122–154% apparent specimen recovery. Conversely, the results of the inter-assay analysis are shown in Table 2. For FeTAFC levels at medium (250 ng/mL) and high (1000 ng/mL) concentrations, the %CVs were 16.4% and 9.5%, respectively. For the low concentration (63 ng/mL), the %CV was 25.1%. The inter-assay recovery specimen range was 108–158%.

### 3.3. FeTAFC Detection in Human Urine Matrix Is Comparable to an ELISA Diluent Using the One-Step FeTAFC ELISA

A comparison of the standard curves of absorbance versus concentration (Figure 1c) of the urine matrix to the diluent control gave near identical results, whereby an R^2^ score of 0.9302 in comparison to a score of 0.9098 for the ELISA diluent was observed. An absorbance range (mean ± standard deviation (SD)) of 1.431 ± 0.071 to 0.033 ± 0.003 versus 1.356 ± 0.05 to 0.031 ± 0.003 was obtained for 0–1000 ng/mL FeTAFC detection in ELISA diluent compared to human urine. Comparisons of twelve urine samples from healthy individuals to the urine matrix standard curve (Figure 1c) (x = 10^((y − 1.301)/−0.4587)^) showed that an increase in FeTAFC concentration led to a decrease in signal (Table 3), as predicted. The mean (±SD) basal FeTAFC concentration in urine was 2.7 ± 2.2 ng/mL and the average recovery of spiked FeTAFC was 82, 114, and 56% for 63, 250, and 1000 ng/mL, respectively, indicating assay functionality (Table 3).

### 3.4. The FeTAFC ELISA Is Specific for FeTAFC

**Figure 2:**
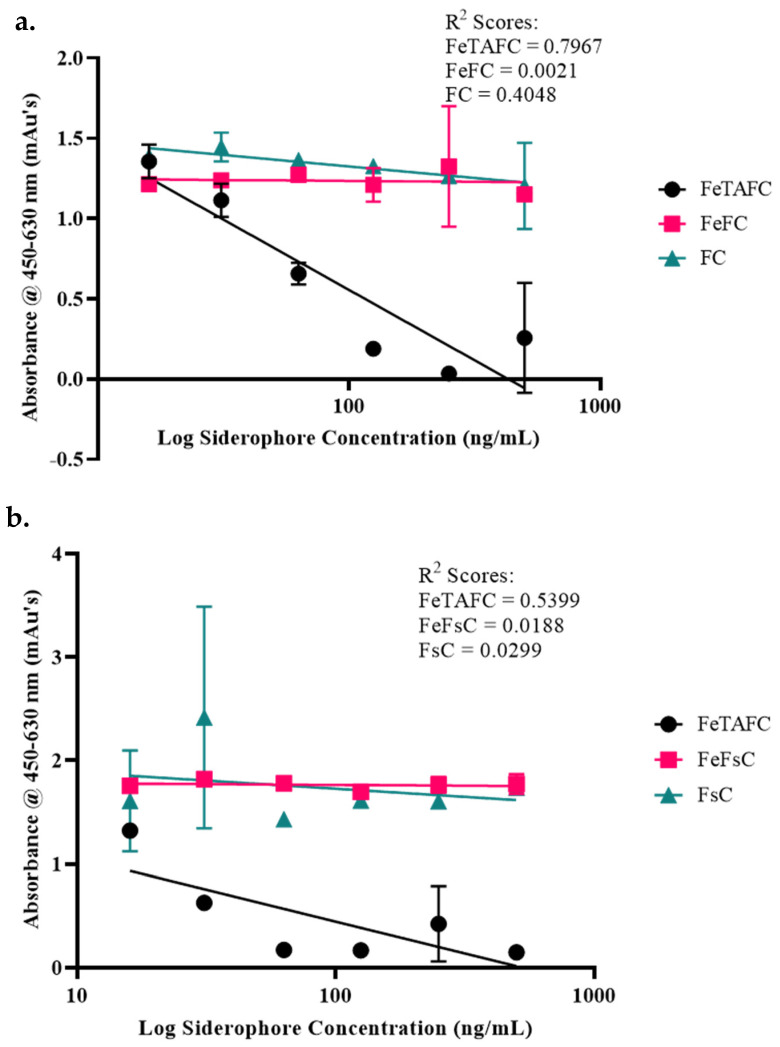

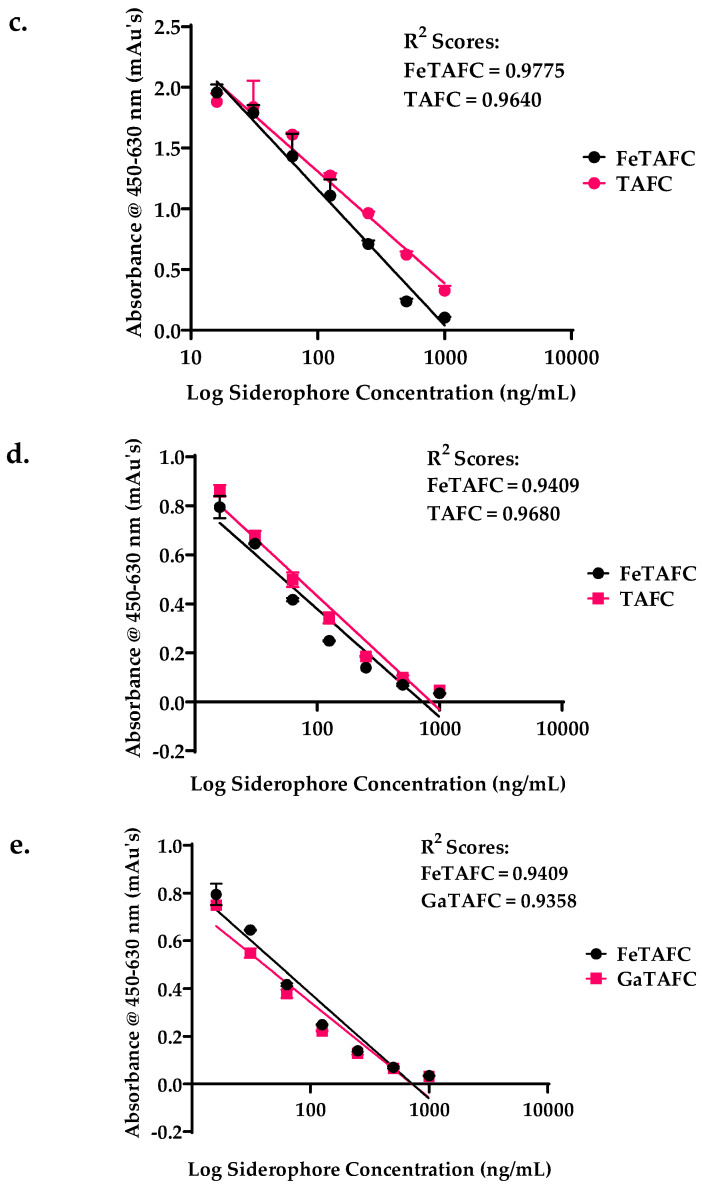
The specificity of the FeTAFC ELISA was shown by analysing a titre of FeTAFC in comparison to (**a**) FeFC and FC and (**b**) FeFsC and FsC. Compared to the standard curve of FeTAFC, an increase in the concentration of FeFC or FC did not lead to a proportional change in absorbance. As with FeFC and FC, no change in the absorbance was seen corresponding to the increased concentration of FeFsC and FsC. This shows that the IgG [anti-FeTAFC] is highly specific for FeTAFC. The specificity of the FeTAFC ELISA was tested by assessing the ability of the (**c**) IgG [anti-FeTAFC] and (**d**) IgG [anti-FeTAFC]-HRP conjugate to detect unbound TAFC, as well as (**e**) the IgG [anti-FeTAFC]-HRP conjugate to detect GaTAFC.

Comparisons of titres of FeTAFC to titres of FeFC, FC, FeFsC, and FsC (16–1000 ng/mL) using the two-step FeTAFC ELISA [26] showed that no decrease in absorbance was seen with an increase in the concentration of any of the other *A. fumigatus* siderophores tested (Figure 2a,b), which confirmed the specificity of IgG [anti-FeTAFC] for this siderophore. Analysis of FeTAFC or TAFC titres (16–1000 ng/mL) on the two-step FeTAFC ELISA showed that there was a decrease in absorbance with increased TAFC; however, it was not to the same extent as that of FeTAFC’s standard curve (Figure 2c). The one-step FeTAFC ELISA, however, showed almost identical results for the TAFC and GaTAFC titre as the FeTAFC (Figure 2d,e). Based on this data, we hypothesise that TAFC is chelating residual media or HRP-derived Fe^3+^ and is detected as FeTAFC.

### 3.5. IgG [Anti-FeTAFC] Disrupts the Growth of A. fumigatus ΔsidD

**Figure 3:**
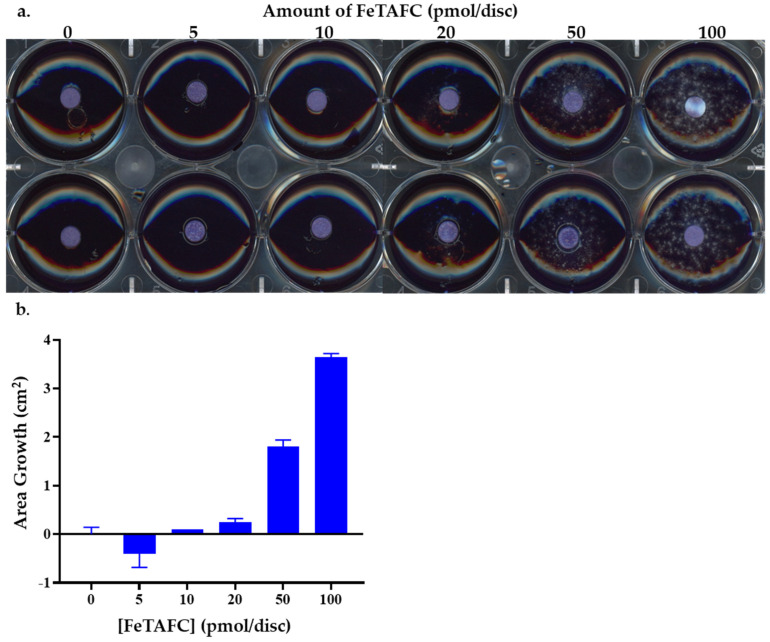

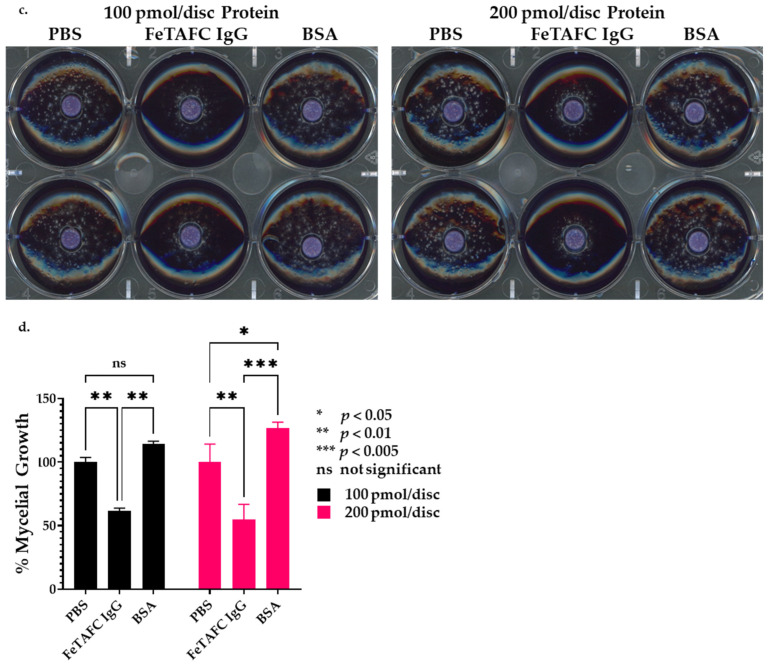
Exogenous FeTAFC enables growth of *A. fumigatus* Δ*sidD* at 50 conidia/well in the presence of different concentrations of FeTAFC (0–100 pmol/disc). (**a**) Growth of *A. fumigatus* Δ*sidD* (as shown by speckled mycelial growth) on iron-free solid agar with different amounts of FeTAFC dyed with a blue contrast dye. (**b**) The area of growth on the agar plates was analysed by imageJ contrast area analysis. (**c**) *A. fumigatus* Δ*sidD* on iron-free solid agar with 50 pmol/disc FeTAFC and 100 and 200 pmol/disc IgG [anti-FeTAFC], as well as controls, dyed with a blue contrast dye. IgG [anti-FeTAFC] impedes growth of *A. fumigatus* Δ*sidD* at 50 conidia/well with 50 pmol/disc FeTAFC and IgG [anti-FeTAFC] at 100 and 200 pmol/disc. Negative controls via addition of PBS and protein control with 100 and 200 pmol/disc were added instead of IgG [anti-FeTAFC] for comparison. (**d**) The percentage growth of *A. fumigatus* Δ*sidD* with FeTAFC and IgG [anti-FeTAFC]. At 100 pmol/disc, there is a significant decrease in growth with IgG [anti-FeTAFC] in comparison to the PBS (*p* = 0.0073) and BSA (*p* = 0.0003) controls. There is no significant difference between the PBS and BSA controls (*p* = 0.2417). At 200 pmol/disc, there is a significant decrease in growth with IgG [anti-FeTAFC] in comparison to the PBS (*p* = 0.0032) and BSA (*p* = 0.0003) controls. There is a significant difference between the PBS and BSA controls (*p* = 0.0357); however, it is a significant increase in growth with BSA, rather than a decrease.

The growth of the *A. fumigatus ΔsidD* was inhibited by the presence of IgG [anti-FeTAFC]. It was first confirmed that at a conidial concentration of 50 conidia/well, *A. fumigatus* Δ*sidD* (incapable of forming the ester bonds between *N*^5^-*cis*-anhydromevalonyl-*N*^5^-hydroxy-L-ornithine residues essential for the production of FsC and therefore TAFC) under iron-deplete conditions, could not grow without the addition of FeTAFC (Figure 3a,b). Supplementation with a titre of FeTAFC concentrations (5–100 pmol/disc) showed that 50 pmol/disc FeTAFC was needed to restore this growth (Figure 3a,b). When the 50 pmol/disc FeTAFC was preincubated with either IgG [anti-FeTAFC], BSA, or PBS (100 and 200 pmol/disc), fungal growth was significantly inhibited in comparison to PBS (*p* = 0.0073 at 100 pmol/disc and *p* = 0.0032 at 200 pmol/disc IgG [anti-FeTAFC]) and BSA (*p* = 0.0003 at 100 pmol/disc and *p* = 0.0003 at 200 pmol/disc IgG [anti-FeTAFC]). BSA produced an increase in the growth of *A. fumigatus* Δ*sidD* (Figure 3c,d). These findings underpinned the subsequent assessment of *A. fumigatus* wild-type growth inhibition studies by IgG [anti-FeTAFC].

### 3.6. Growth of the A. fumigatus Is Impeded by Incubation with Higher Amounts of IgG [Anti-FeTAFC]

**Figure 4:**
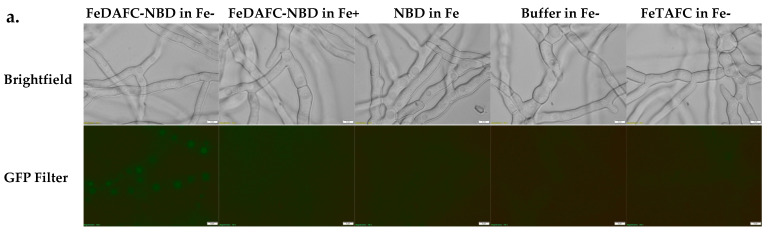

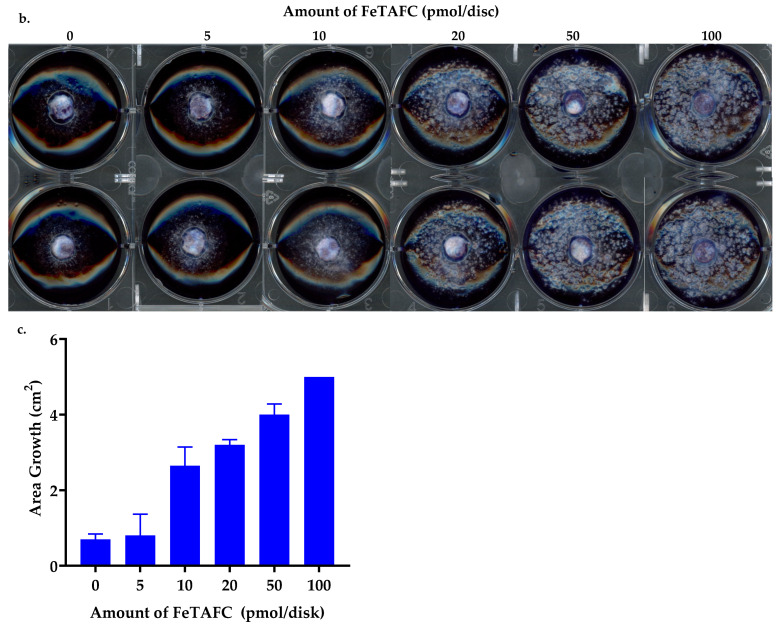

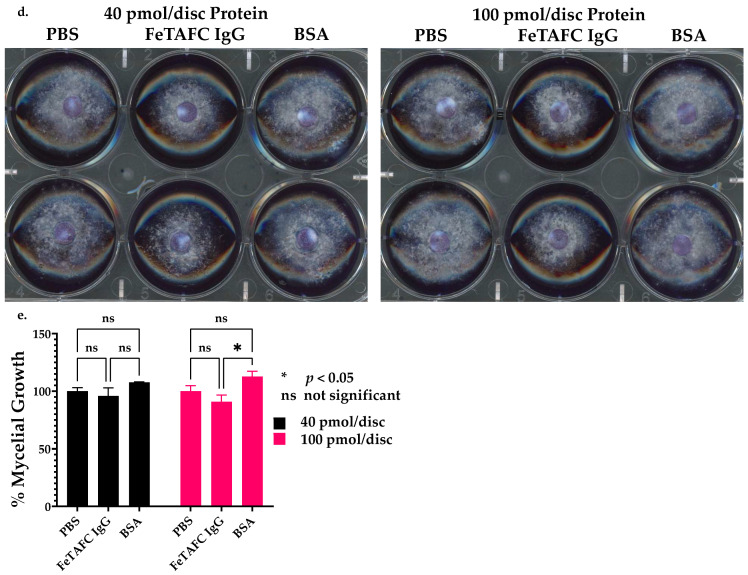
Siderophore uptake by *A. fumigatus*. (**a**) Uptake of fluorescent FeTAFC analogue (FeDAFC-NBD) by *A. fumigatus*. *A. fumigatus* was grown in MM with (Fe+) and without (Fe-) iron, followed by incubation with DAFC-NBD or controls at an equivalent concentration for 1 h. After washing, mycelia were viewed by fluorescent microscopy. Fluorescence was observed to localise to vacuoles after incubation with FeDAFC-NBD only when grown without iron. (**b**) Growth enhancement of *A. fumigatus* wild-type on iron-free agar with the addition of different amounts of FeTAFC, as assessed following staining with a contrast dye visually and (**c**) using imageJ analysis. (**d**) Effect of IgG [anti-FeTAFC] on growth of *A. fumigatus* wild-type at 50 conidia/well with 20 pmol/disc FeTAFC and IgG [anti-FeTAFC] at 40 and 100 pmol/disc. Negative controls with the addition of PBS and protein of 40 and 100 pmol/disc were added in the place of IgG [anti-FeTAFC] for comparison. Comparison of the growth with and without IgG [anti-FeTAFC] were analysed (**d**) visually and (**e**) graphically. At 40 pmol/disc, there was a decrease in growth with IgG [anti-FeTAFC] in comparison to the PBS (*p* = 0.7908) and BSA (*p* = 0.1276) controls, but it was not significant. There was no significant difference between the PBS and BSA controls (*p* = 0.3901). At 100 pmol/disc, there was a decrease in growth with IgG [anti-FeTAFC] in comparison to the PBS (*p* = 0.2720) control, which was not significant, while the BSA (*p* = 0.0105) control was significant. There was no significant difference between the PBS and BSA controls (*p* = 0.1020).

Siderophores are acquired by *A. fumigatus* specifically under iron-limiting growth conditions. To confirm this, fluorescence microscopy analysis assessed the uptake of this FeDAFC-NBD (Supplementary Figure S3) by *A. fumigatus* mycelia. Vacuoles of hyphae incubated with FeDAFC-NBD only showed fluorescence when grown in iron-free conditions (Figure 4a). This shows that FeDAFC, an immunologically indistinguishable FeTAFC analogue (Figure 1 and Figure 2), is selectively taken up by *A. fumigatus* when conjugated to other molecular species.

Unlike *A. fumigatus* Δ*sidD*, the growth of *A. fumigatus* wild-type under iron-limiting conditions was not completely prevented due to the production of siderophores at a conidial concentration of 50 conidia/well, with an average of 0.7 cm^2^ of growth observed (Figure 4b,c). However, supplementation with FeTAFC increased the growth of *A. fumigatus* wild-type, with 20 pmol/disc yielding sufficient growth for a decrease to be recorded (Figure 4b,c). Preincubation of 20 pmol/disc FeTAFC with IgG [anti-FeTAFC] was seen to lead to a decrease in growth at both 40 (*p* = 0.7908) and 100 pmol/disc (*p* = 0.2720) in comparison to the PBS control (Figure 4d,e). BSA was seen to increase the growth at both concentrations. There was a significant difference between BSA and the IgG [anti-FeTAFC] (*p* = 0.0105) at 100 pmol/disc (Figure 4d,e), indicating growth inhibition due to restricted FeTAFC uptake.

### 3.7. IgG [Anti-FeTAFC] Impedes the Growth of A. fumigatus in Microwell Liquid Culture and Alters the Siderophore Content of Liquid Culture Supernatants

**Figure 5:**
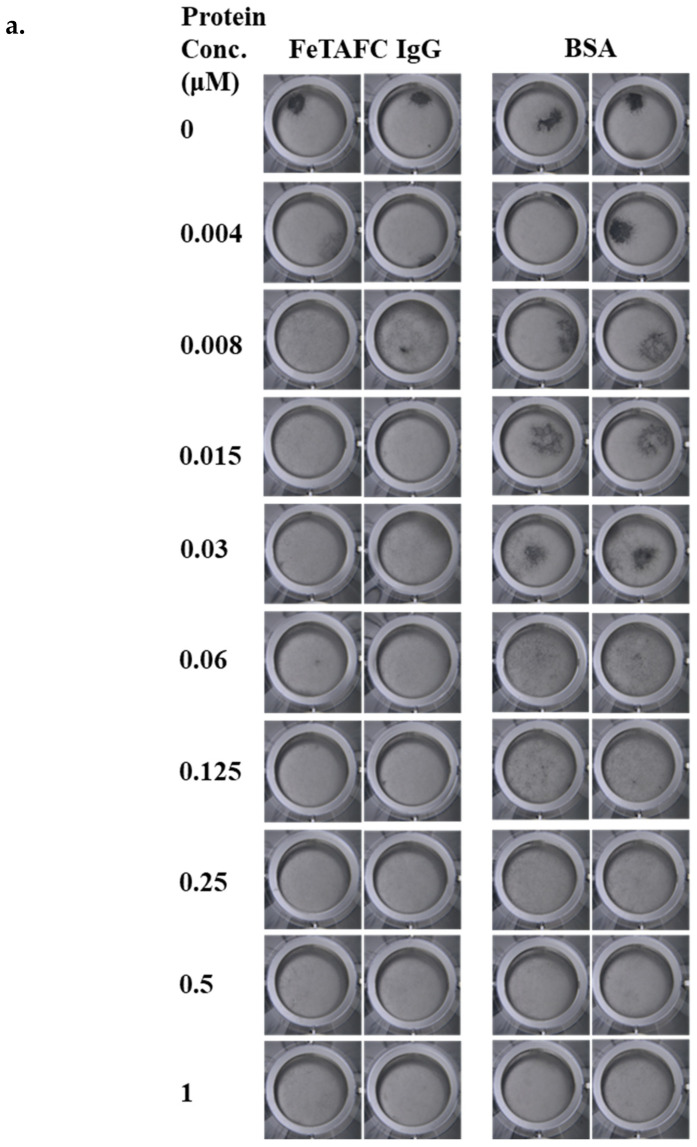

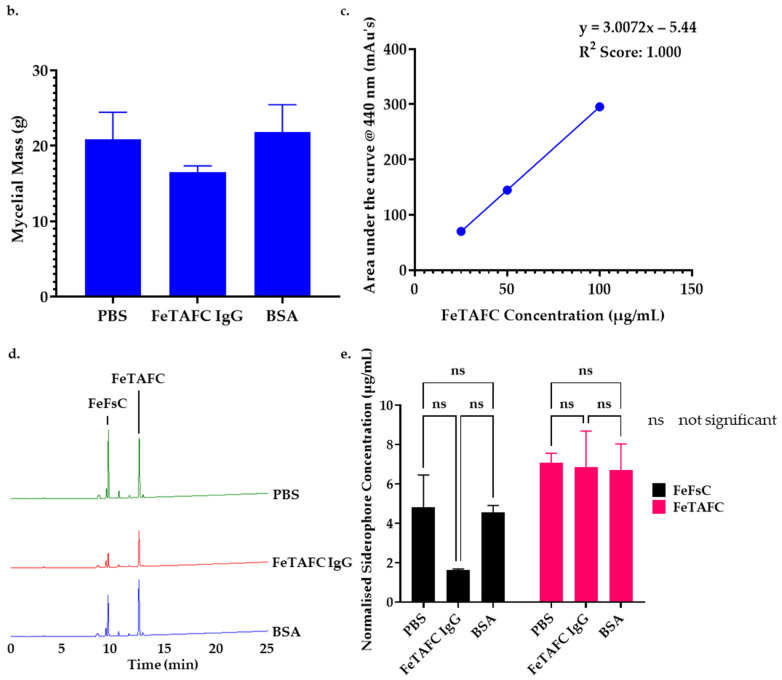
IgG [anti-FeTAFC]’s impact on fungal growth and siderophore production. (**a**) IgG [anti-FeTAFC] impairs *A. fumigatus* wild-type growth in microwells compared to BSA controls (0–1 µM protein concentration). *A. fumigatus* is seen as clusters of dark mycelia in the wells, whereby assays were carried out in iron-free liquid media in microwells (200 μL) and imaged using microscopy. (**b**) Analysis of effects of IgG [anti-FeTAFC] on the growth of *A. fumigatus* in liquid culture and siderophore content of the *A. fumigatus* supernatant. The wet mycelial mass of *A. fumigatus* after 48 h incubation with IgG [anti-FeTAFC] (0.015 μM). (**c**) Standard curve of RP-HPLC AUC (mAu’s) at 440 nm for FeTAFC. (**d**) RP-HPLC analysis at 440 nm of supernatants following *A. fumigatus* post-incubation with PBS, IgG [anti-FeTAFC], or BSA. (**e**) The concentration of FeFsC and FeTAFC (μg/mL) normalised to the mycelial mass of *A. fumigatus* under each condition. The normalised concentration of FeFsC is seen to be similar in the PBS and BSA controls (*p* = 0.9719) but lower in the IgG [anti-FeTAFC] samples in comparison to the PBS (*p* = 0.0741) and BSA (*p* = 0.0989) controls. These differences are not significant, but they are lower. The normalised FeTAFC concentration, however, is similar between the controls (*p* = 0.9421) and between the IgG [anti-FeTAFC] treatment and the PBS (*p* = 0.9747) and BSA (*p* = 0.9928) controls.

Visual analysis of an *A. fumigatus* microwell growth assays revealed that after 24 h at 37 °C, *A. fumigatus* growth was reduced by 0.015 μM IgG [anti-FeTAFC] in comparison to the controls (Figure 5a). A growth decrease was seen with 0.5 μM BSA, but only at a 33.3× higher concentration (Figure 5a). In larger cultures, the wet mass of *A. fumigatus* mycelia after 48 h at 37 °C was decreased in the presence of 0.015 μM IgG [anti-FeTAFC] in comparison to the PBS control, whereas BSA at the same concentration caused increased growth (Figure 5b). After the post-culture addition of iron (to ferrate any siderophores produced for detection at 440 nm) and the removal of BSA or antibody from the supernatant using 50 kDa spin filters, RP-HPLC analysis showed a decrease in the concentration of FeFsC in the IgG [anti-FeTAFC] supernatants compared to the PBS and BSA controls when accounting for weight (Figure 5c–e). This was unexpected and may indicate that SidG-mediated acetylation of FeFsC is increased in *A. fumigatus* consequent to extracellular FeTAFC complexation by the IgG [anti-FeTAFC]. Therefore, it is likely that a higher concentration of antibodies would be needed to achieve full inhibition of *A. fumigatus* growth.

## 4. Discussion

New diagnostic strategies for IA are needed due to the low specificity and sensitivity of many of the current strategies for diagnosing *A. fumigatus* infection, and the previous detection of TAFC/FeTAFC in the urine of IA patients highlighted its use as a potential biomarker [34]. To this end, a rapid, specific, and sensitive FeTAFC ELISA for the detection of FeTAFC in urine was developed using IgG [anti-FeTAFC] to overcome issues with technology accessibility associated with mass spectrometry instrumentation. Moreover, the IgG [anti-FeTAFC] was also shown to impede *A. fumigatus* growth in vitro and alter the supernatant FsC/TAFC ratio under iron-free growth conditions. This infers future immunotherapeutic evaluation of IgG [anti-FeTAFC], since iron limitation exists in vivo and TAFC is more abundant than FsC. Overall, the work presented herein illustrates the potential opportunity for IgG [anti-FeTAFC] to open new research directions for aspergillosis detection and treatment.

Immunoblot analysis confirmed that the antibody conjugate could detect immobilised FeDAFC-BSA at >1.5 ng. ELISA analysis revealed that the absence of free specimen FeTAFC resulted in antibody conjugate binding to immobilised FeDAFC-BSA and colour change was observed with the addition of TMB, whereas the addition of free FeTAFC, up to 1000 ng/mL, caused a subsequent reduction in absorbance. Therefore, this antibody conjugate was viable for use in a one-step ELISA for FeTAFC immunodetection. Regarding assay precision, while at the lower FeTAFC concentration tested (63 ng/mL), variation was somewhat high for both the intra and inter-assay precision, at medium (250 ng/mL) and high (1000 ng/mL) concentrations, the observed %CV values were below 20% and within an acceptable range. The use of IgG [anti-FeTAFC] for the direct detection of urinary FeTAFC has not previously been explored. However, it has been shown that TAFC is present in urine of either rodent models or human patients, is associated with IA infection, and can be detected using MS analysis. Fourier transform ion cyclotron (FTICR) MS was used to determine siderophore presence in infected rats with a limit of detection (LOD) of 0.1 ng/mL [19]. TAFC was detected in all infected animal urine samples tested (*n* = 6), with a mean concentration of 370 ± 170 ng/mL. LC-MS was shown to detect TAFC in urine of probable IA patients to a LOD of 0.1 ng/mL [23]. This study mostly normalised TAFC concentrations to creatinine concentration in the urine; however, four serial samples from one probable IA patient were shown to have a concentration range of 28.2–146.8 ng/mL TAFC. These TAFC concentrations in urine were much higher than had been previously seen in the serum of IA patients, which had a mean concentration of 11.6 ng/mL in suspected IA patients (*n* = 76) and 9.7 ng/mL in proven or probable cases (*n* = 14) with a LOD of 1 ng/mL using LC-MS [35]. Given that the equipment necessary for LC-MS analysis is not available in many hospital settings, a more rapid and less labour-intensive method would be more useful in these settings, prompting the implementation of urinary FeTAFC detection by ELISA.

Urine as a matrix for ELISA analysis can lead to assays with low sensitivity due to the pH and ion content of the solution [14,36] and therefore more invasive tests are often used [37]. However, it is possible for assays to have similar sensitivity with urine as other matrices [38]. As such, it was important to ensure that this matrix was suitable for this assay. A comparison of the standard curves of FeTAFC in urine and ELISA diluent revealed minimal difference between the two matrices. This indicates that urine is a viable matrix for the FeTAFC ELISA, as it performs almost identically to the ELISA diluent matrix with low background and has sensitivity down to the range of FeTAFC detection reported in previous TAFC urine studies (Table 3).

Twelve urine samples, collected from healthy volunteers, were analysed using the FeTAFC ELISA, and a mean concentration of 2.7 ng/mL was seen with a range of 0.9–9 ng/mL FeTAFC. These samples were then spiked with FeTAFC at low (50 ng/mL), medium (250 ng/mL), and high (1000 ng/mL) concentrations. The low, medium, and high concentration samples yielded mean concentrations of 41.1, 286.5, and 558.8 ng/mL, respectively. Overall, these data confirm possible low levels of TAFC in normal human urine and that the FeTAFC ELISA can detect FeTAFC in spiked urine specimens, which indicates the capability to detect endogenous FeTAFC in clinical specimens. It is notable that the lowest calibrator on the standard curve (16 ng/mL) is higher that the limit of detection seen with MS analysis, although it is still lower than the levels being detected in the urine of probable IA patients [19,23]. Further analysis will be needed to ensure that FeTAFC can be detected in clinical samples using the one-step FeTAFC ELISA. FeTAFC has previously been detected in urine from healthy controls by capillary electrophoresis electrospray ionisation mass spectrometry (CE-ESI-MS) [23], and future work is required to understand this phenomenon. We, and others [23], speculate that this is likely due to dietary origin or environmental *A. fumigatus* conidial exposure, which will be explored in future work.

It was important to assess the specificity of the FeTAFC ELISA to rule out any detection of other siderophores. *A. fumigatus* produces three other siderophores, FsC, FC, and hydroxy–FC, from the same precursor molecule that is used to make TAFC, L-ornithine [32]. FsC comprises the same three *N*-*cis*-anhydromevalonyl-N-hydroxy-L-ornithine subunits as TAFC, with the only structural difference to TAFC being the unacetylated amine groups. Antibodies can sometimes detect multiple molecules with a similar structure; for example, analysis of a fentanyl ELISA showed that a number of analogues of the drug were detected [39], and an ELISA for the detection of the liver protein vitellogenin in *Pleuronectes titulus* showed reactivity with other plasma proteins [40]. Low specificity in assays can lead to false positives. To ensure the same was not seen with the FeTAFC ELISA, two other *A. fumigatus* siderophores were analysed, FC and FsC. Detection of FeTAFC in comparison to FeFC, FC, FeFsC, and FsC showed that the IgG [anti-FeTAFC] did not detect any of these comparator siderophores, as no decrease in absorbance was seen with an increase in concentration. Even though these siderophores have similar structures to FeTAFC, the IgG [anti-FeTAFC] is highly specific for FeTAFC. Previously, the IgG [anti-FeTAFC] was analysed for its ability to detect FsC [26]. This analysis was identical to that seen with the IgG [anti-FeTAFC] herein, with no activity against FsC.

As the IgG [anti-FeTAFC] had been raised against FeDAFC-KLH, all analysis was carried out on FeTAFC; however, it was possible that the antibody would detect unferrated TAFC or TAFC bound to other metals. We rationalised that it is preferable for the IgG [anti-FeTAFC] to either detect TAFC and FeTAFC collectively or FeTAFC alone, as opposed to exhibiting a higher affinity or specificity for TAFC. TAFC has been shown to chelate gallium [41,42,43], and so GaTAFC was also detected by the FeTAFC ELISA. Previously, antibodies against the siderophore enterobactin were shown to have lower activity against unferrated enterobactin in comparison to ferrated enterobactin and were shown to detect enterobactin bound to other metals such as magnesium and copper, but not zinc [44].

Determining if the FeTAFC ELISA detects TAFC and GaTAFC proved to be difficult, as iron is ubiquitous in the environment; however, all possible precautions were taken to ensure that iron contamination was minimised. Analysis showed conflicting results with the one- and two-step ELISAs. The one-step ELISA yielded identical results for FeTAFC and TAFC detection, while the two-step FeTAFC ELISA showed superior detection of FeTAFC at higher concentrations, though at lower TAFC/FeTAFC concentrations the detection was equivalent—possibly due to TAFC acquisition of free Fe^3+^ in diluents/blocking agents. Thus, at higher TAFC concentrations, there may not have been enough environmental iron to fully ferrate the siderophore, resulting in reduced detection of the unferrated TAFC compared to FeTAFC. Conversely, the antibody conjugate may contribute iron (due to HRP presence) which may account for the identical results for TAFC and FeTAFC detection by the one-step FeTAFC ELISA. Similarly, the one-step FeTAFC ELISA showed similar results for FeTAFC and GaTAFC. This is likely due to the antibody conjugate recognising metal-bound TAFC as either FeTAFC or GaTAFC.

Studies on the generation of siderophore-specific antibodies are very limited and generally focus on bacterial, rather than fungal, siderophores. One of the first examples of monoclonal antibodies against siderophores was against pseudobactin, a siderophore produced by *Pseudomonas putida*. These antibodies were used in an ELISA with sensitivity to 5 × 10^−12^ mol of pseudobactin [45]. Faecal samples from mice immunised with enterobactin, a bacterial siderophore, conjugated to an immunogenic protein cholera toxin subunit B, were shown to contain antibodies that detected immobilised enterobactin–PEG_3_-biotin conjugate on an ELISA plate [46,47]. These studies focused on the effect of immunisation with these conjugates on the colonisation of gut *Salmonella* species and *Escherichia coli*, which were both reduced by siderophore-conjugate immunisation, and did not develop this ELISA further to show sensitivity of enterobactin detection. Monoclonal antibodies against an enterobactin–KLH conjugate were used to develop a competitive ELISA with sensitivity to 3.1 × 10^−10^ M enterobactin [48]. However, none of the antibodies previously developed against siderophores detected a fungal siderophore.

Due to the rise in antimicrobial resistance, there is a need for new antimicrobial agents. To this end, we investigated the use of the IgG [anti-FeTAFC] to inhibit *A. fumigatus* growth. This strategy targeted the fungal siderophore FeTAFC, which is essential for the virulence of *A. fumigatus* [24]. Firstly, we confirmed that *A. fumigatus* was capable of siderophore uptake only under iron-limiting conditions using fluorescent FeDAFC uptake. This expanded our previous demonstration of fluorescent FeFsC-NBD uptake [33] and complemented that of FeDAFC-Cy5 and NBD fluorescent conjugates by Pfister et al. [43,49]. On solid media, growth inhibition was more significant with *A. fumigatus ΔsidD* than *A. fumigatus* wild-type. This is likely because *A. fumigatus ΔsidD* is incapable of producing either the precursor molecule FsC or TAFC [50] and so the only FeTAFC available is as provided experimentally. With *A. fumigatus* wild-type, a higher concentration of IgG [anti-FeTAFC] would be needed to bind or complex endogenously produced TAFC (after iron chelation); however, concentrations as low as 100 pmol IgG [anti-FeTAFC] showed inhibition, even if it was not significant. In liquid media, there was an inhibition of growth in comparison to the BSA control at low concentrations and an impact on the siderophore production. FeFsC was present at approximately 50% in the IgG [anti-FeTAFC]-treated conditions in comparison to the controls. As FeTAFC and not FeFsC is essential for the virulence of *A. fumigatus* [51], it is likely that SidG, responsible for the acetylation of FsC [50], was upregulated in order to compensate for the binding of FeTAFC by IgG [anti-FeTAFC].

In conclusion, the use of IgG [anti-FeTAFC] in a one-step FeTAFC ELISA gave reproducible, specific results and is suitable for use to specifically detect FeTAFC in human urine. The FeTAFC ELISA, with further testing and validation using clinical specimens, including well-defined limits of detection, could be a useful tool in the diagnosis of IPA. It is also possible that the antibody, subject to extensive evaluation in IA infection model systems (e.g., immunocompromised mice), may have potential therapeutic application against *A. fumigatus*, as it recognises FeTAFC, a siderophore essential for the growth and virulence of *A. fumigatus*. This work, including Supplementary Materials [32,52,53], therefore provide initial evidence for the application of IgG [anti-FeTAFC] regarding both the detection and possible growth reduction in *A. fumigatus*.

## Figures and Tables

**Table 1:** The intra-assay precision of the one-step FeTAFC ELISA determined using % coefficient of variation (%CV) of samples (*n* = 5) assayed on the same ELISA plate. Samples were analysed in duplicate at low (L), medium (M) and high (H) concentrations, where L = 63 ng/mL, M = 250 ng/mL, and H = 1000 ng/mL FeTAFC. The absorbances were compared to the standard curve in Figure 1b and the %CVs were calculated.

FeTAFC (ng/mL)					
Test	Mean Conc.	SD	%CV	Range	*n*
L	83	35	42	50.8–138.4	5
M	305	77	25	200.1–397.6	5
H	1545	202	13	1286.6–1799	5

**Table 2:** The inter-assay precision of the one-step FeTAFC ELISA determined using %CV of samples over eight different assays. Samples were analysed in duplicate at low (L), medium (M) and high (H) concentrations, where L = 63 ng/mL, M = 250 ng/mL, and H = 1000 ng/mL FeTAFC. The absorbances were compared to the standard curve in Figure 1b and the %CVs were calculated.

FeTAFC (ng/mL)					
Test	Mean Conc.	SD	%CV	Range	*n*
L	97.8	25.1	25.1	54.7–137.5	8
M	264.8	43.4	16.4	191.0–321.0	8
H	913.9	87.1	9.5	794.6–1042.8	8

**Table 3:** Urine samples were collected from twelve healthy individuals. Each sample was spiked with FeTAFC at low (L), medium (M), and high (H) concentrations. The FeTAFC concentrations used were L = 50 ng/mL, M = 250 ng/mL, and H = 1000 ng/mL. The twelve samples were analysed in duplicate, both unspiked and at each concentration, using the one-step FeTAFC ELISA alongside controls in ELISA assay buffer.

	Unspiked		L		M		H	
Test	Absorbance	ng/mL	Absorbance	ng/mL (% R)	Absorbance	ng/mL (% R)	Absorbance	ng/mL (% R)
ELISA Assay	1.308	1.0	0.518	50.9 (101.9)	0.131	355.4 (142.1)	0.034	579.8 (58.0)
Sample 1	1.277	1.1	0.571	39.1 (78.3)	0.151	322.2 (128.9)	0.031	588.6 (58.9)
Sample 2	1.121	2.5	0.652	26.1 (52.1)	0.219	228.5 (91.4)	0.048	540.4 (54.0)
Sample 3	1.284	1.1	0.637	28.1 (56.2)	0.180	278.6 (111.4)	0.041	559.7 (56.0)
Sample 4	0.863	9.0	0.452	70.9 (141.9)	0.141	338.8 (135.5)	0.036	574.0 (57.4)
Sample 5	1.223	1.5	0.544	44.8 (89.6)	0.162	304.2 (121.7)	0.037	571.1 (57.1)
Sample 6	1.251	1.3	0.547	44.0 (88.1)	0.163	303.4 (121.4)	0.040	562.6 (56.3)
Sample 7	1.117	2.5	0.602	33.4 (66.8)	0.227	219.5 (87.8)	0.047	541.8 (54.2)
Sample 8	1.313	0.9	0.657	25.3 (50.7)	0.212	237.2 (94.9)	0.050	533.7 (53.4)
Sample 9	1.135	2.3	0.589	35.8 (71.5)	0.173	288.5 (115.4)	0.041	558.3 (55.8)
Sample 10	1.094	2.8	0.509	53.3 (106.6)	0.135	349.2 (139.7)	0.033	581.2 (58.1)
Sample 11	0.982	5.0	0.572	38.8 (77.7)	0.180	277.9 (111.2)	0.043	554.1 (55.4)
Sample 12	1.125	2.4	0.507	54.0 (107.9)	0.172	290.0 (116.0)	0.048	540.4 (54.0)
Mean	1.148	2.7	0.570	41.1 (82.3)	0.176	286.5 (114.6)	0.041	558.8 (55.9)
SD	0.127	2.179	0.060	12.802	0.029	39.770	0.006	16.840

## Data Availability

The original contributions presented in this study are included in the article/Supplementary Material.

## Supplementary Materials

The following supporting information can be downloaded at: https://www.mdpi.com/article/10.3390/jof12050342/s1. Figure S1. Growth assay was established using *A. fumigatus* Δ*sidD* streaked on MM without iron. Discs (white) supplemented with a titre of FeFsC amounts (5–100 pmol/disc) were subsequently added and plates were incubated at 37 °C for 48 h. Exogenous supplementation of FeFsC (20, 50, and 100 pmol) was found to restore growth of *A. fumigatus* Δ*sidD* at 48 h. Figure S2. Growth assays were established using *A. fumigatus* Δ*sidD* grown on MM without iron with discs supplemented with (i) FeFsC only, (ii) FeFsC mixed with anti-FeFsC IgM (IgM), or (iii) FeFsC mixed with GST-GtmA (negative control, at an equivalent protein concentration to the IgM). Plates were subsequently grown for a. 48 h, or b. 96 h. Anti-FeFsC IgM inhibits FeFsC-mediated growth restoration of *A. fumigatus* Δ*sidD* at a ratio of 50 pmol IgM/20 pmol FeFsC per disc after 96 h of growth (highlighted in red). Figure S3. Fluorescent FeDAFC, synthesised from FeFsC precursor, is acquired by *A. fumigatus* specifically under iron-deplete conditions. a. RP-HPLC analysis of the reaction of FeFsC with sulfo-NHS-acetate, with the intensity of the peaks for MAFC, DAFC and TAFC shown. The reaction was carried out with a 2, 4 and 8 molar excess of sulfo-NHS-acetate over FeFsC, in order to determine the optimum molar ratio to produce DAFC. b. LC-MS/MS identification of DAFC-NBD. DAFC-NBD reaction was analysed by LC-MS/MS. (A) MS (extracted ion chromatogram for *m/z* 1139–1142) spectrum shows the detection of DAFC-NBD as a singly charged ion (M: 1139.4, [M+H]+: observed *m/z* 1140.4; expected *m/z* 1140.4). MS2 spectrum shows the fragmentation of precursor ion. Increments of approx. +1.0 in observed *m/z* are indicative of C13 isotope incorporation in a singly charged ion. Table S1. Volumes of FeFsC, SNA and DMF required for the various reaction ratios.
