# Supplementary material for: Potential Diagnostic and Research Applications of a Recombinant Antibody Directed Against Ferrated Triacetylfusarinine C from Aspergillus fumigatus
Source: J Fungi (Basel). 2026 May 6;12(5):342. doi: 10.3390/jof12050342 (PMC13209087; doi:10.3390/jof12050342)
Supplement: Supplementary file 1 [file jof-12-00342-s001.zip › jof-4193787-supplementary.pdf]

Potential Diagnostic and Research Applications of a Recombinant Antibody Directed Against Ferrated Triacetylfusarinine C from *Aspergillus fumigatus*

Marie Dwyer [1,†], Rebecca A. Owens [1], Claudia Garcia Revuelto [1], Kieran G. Walshe [2], Cathal M. Murphy [1], Nicola M. Moloney [1] and Sean Doyle [1,*]

[1] Department of Biology, Maynooth University, W23 XY3X Maynooth, Ireland
[2] Accuplex Diagnostics Limited, A85 CD51 Ratoath, Ireland
* Correspondence: sean.doyle@mu.ie; Tel.: +353-1-7083858; Fax: +353-1-7083845
† Current address: STGenetics, Dovea Upper, E41 FH28 Thurles, Ireland.

MATERIALS AND METHODS

Ferrated diacetylfusarinine C (FeDAFC) and fluorescent FeDAFC synthesis

Siderophore acetylation with sulpho-NHS-acetate: Using 100 µL of PEN-7.8 prepared aliquots of ferrated fusarinine C (FeFsC) was resuspended in PEN-7.8 to give a total volume of 1200 µL. 20 µL of the FeFsC was placed in 980 µL of PEN-7.8 (1/50 dilution) and the A440 nm determined. A value of 0.659 was observed which was used to calculate the concentration of FeFsC, 0.93 mg/ml. A series of various molar ratios were set up to identify the optimal reaction ratio for the formation of FeDAFC **(Supplementary Table 1)**. Fluorescent derivatisation of DAFC with succinimidyl 6-(*N*-(7-Nitrobenz-2-Oxa-1,3-Diazol-5-yl)Amino) hexanoate (NBD-X-SE): Prepared FeDAFC was purified by RP-HPLC and suspended in 50:50 0.1 M $NaHCO_3$ pH 8.5: acetonitrile to give a final concentration of 2.2 mM. 2 µmoles of this purified FeDAFC (937 µl) was added to 2 µmoles of NBD-X-SE (77 µl; 26 mM in DMSO). The reaction was left to incubate for 1 h at room temperature in the dark with shaking. RP-HPLC and LC-MS analysis was used to confirm the formation of DAFC-NBD.

Uptake of FeDAFC-NBD by A. fumigatus

Conidia (10^3 / ml) of *A. fumigatus* ATCC 46645 were inoculated into minimal media (MM) with and without iron in a 1 ml/ well in 12 well plates. Sterilised coverslips were used on each well. After 20 h of growth at 30 °C static, supernatants were removed and mycelia were washed with fresh cognate media four times. MM with and without iron was supplemented with 50 µM FeDAFC-NBD. This MM, along with controls with NBD-X-SE only, siderophore only, or reaction buffer only were filter sterilised and added to 12-well plates. Mycelia were incubated with treatments for 1 h at 37 °C. Supernatants were removed and mycelia were washed with 4-(2-Hydroxyethyl) piperazine-1-ethanesulfonic acid, N-(2-Hydroxyethyl) piperazine -N′-(2-ethanesulfonic acid) (HEPES) Glucose (HG) Buffer. Coverslips were mounted onto slides, fixed with nail varnish, and visualised using a fluorescent microscope (Olympus BX51; GFP filter: Ex/Em 492–495 nm/517–527 nm). All treatments were performed at least $n = 2$ and a minimum of 3 images were taken per replicate.

RESULTS

In vitro assessment of growth inhibitory potential of anti-FeFsC IgM

To investigate the effect of anti-FeFsC IgM [1] on the growth of *A. fumigatus*, an assay strategy was devised using *A. fumigatus* Δ*sidD* [2]. *A. fumigatus* Δ*sidD* is a gene deletion strain for the NRPS, SidD, and is incapable of producing extracellular siderophores FsC and TAFC. Absence of the production of extracellular siderophores was confirmed with spectrophotometric (440 nm) analysis of culture supernatants (data not shown). First, a growth assay was established whereby the application of exogenous FeFsC restored growth of *A. fumigatus* Δ*sidD*. *A. fumigatus* Δ*sidD* was streaked onto MM agar without iron (supplemented with iron chelator, bathophenanthrolinedisulfonic acid (BPS); ensuring only siderophore-mediated iron

uptake was utilised) to which sterile assay discs were added. Subsequently, a titre of FeFSC amounts (5 – 100 pmol) was added to each disc (*n* = 2). Plates were grown at 37 °C for 48 h. Exogenous FeFsC was found to visibly restore the growth of Δ*sidD* at 20, 50, and 100 pmol per disc (**Supplementary Figure 1**).

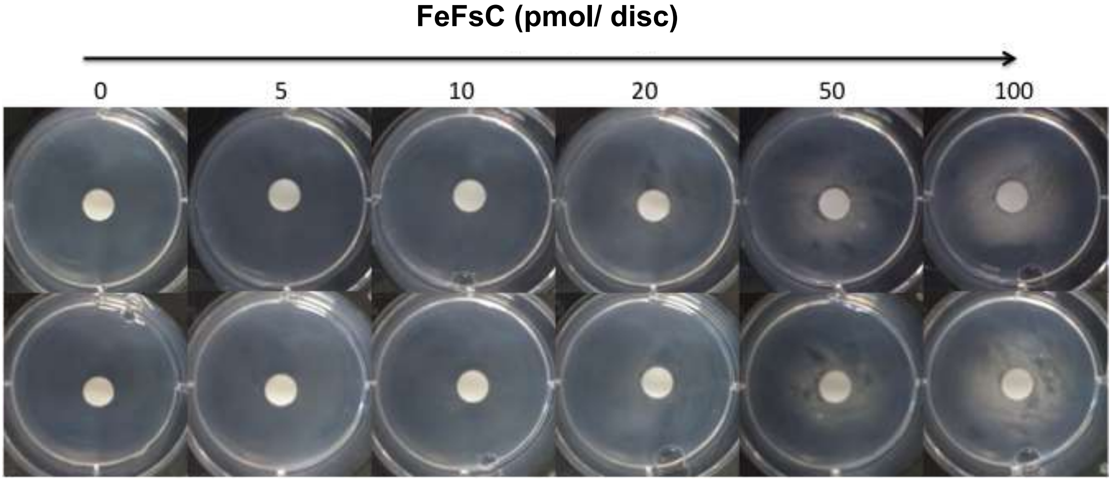

Supplementary Figure S1. Growth assay was established using *A. fumigatus* Δ*sidD* streaked on MM without iron. Discs (white) supplemented with a titre of FeFsC amounts (5 – 100 pmol/ disc) were subsequently added and plates were incubated at 37 °C for 48 h. Exogenous supplementation of FeFsC (20, 50, and 100 pmol) was found to restore growth of *A. fumigatus* Δ*sidD* at 48 h.

Next, the effect of anti-FeFsC IgM on FeFsC-mediated growth restoration was investigated. Prior to addition to discs, FeFsC was pre-incubated with (i) PBS, (ii) anti-FeFsC IgM, or (iii) GST-GtmA fusion protein [3] (at an equivalent protein concentration to the IgM as a negative control) (*n* = 3). Plates were grown at 37 °C for 48 h or 96 h. Anti-FeFsC IgM showed visible inhibition of FeFsC-mediated growth restoration of *A. fumigatus* Δ*sidD* at a ratio of 50 pmol IgM/ 20 pmol FeFsC per disc after 96 h of growth (**Supplementary Figure 2**). As this assay was only qualitative in nature and quantitative analysis was not possible, the reduction in growth was verified by the blind observations of two other individuals.

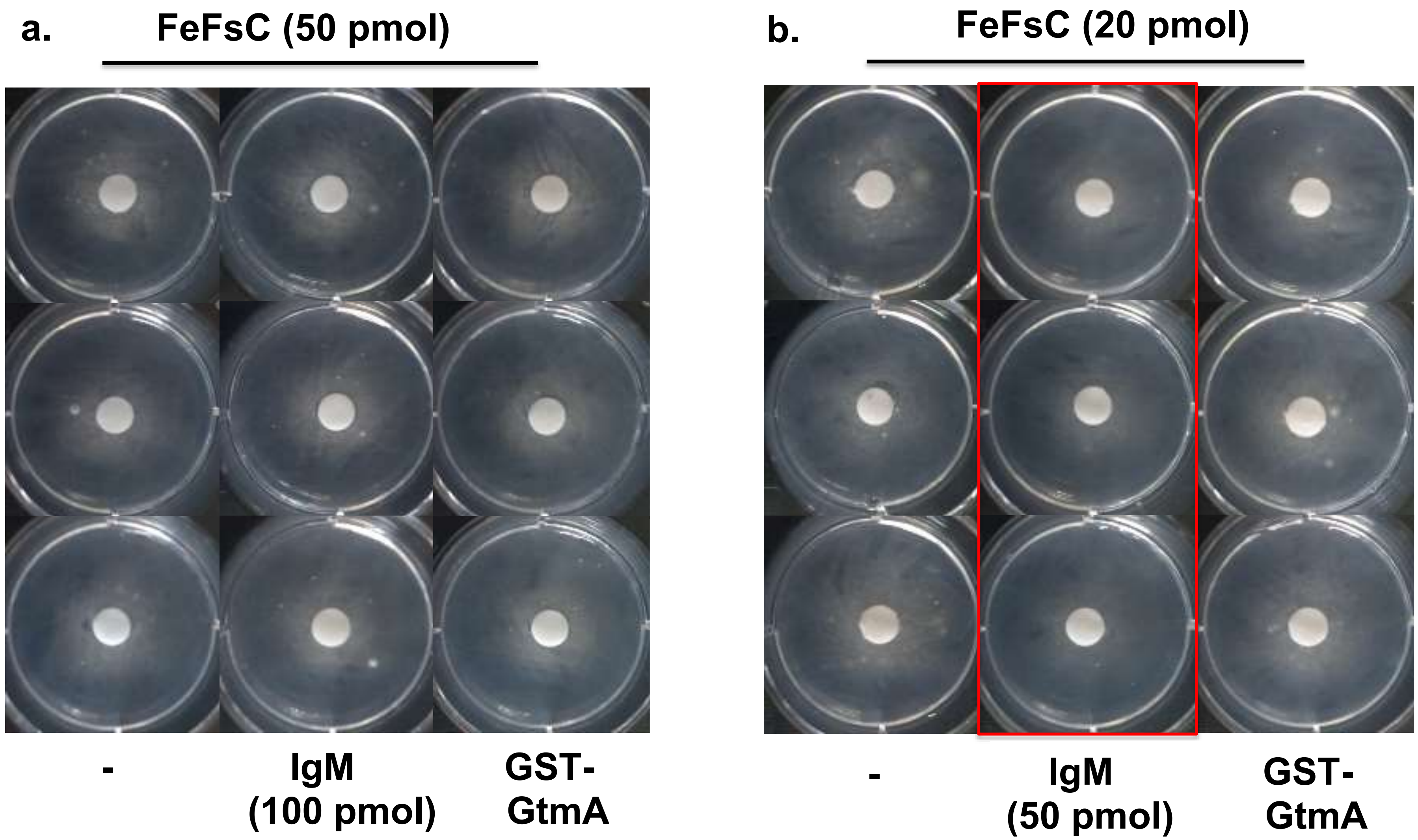
a.
FeFsC (50 pmol)
-
IgM
(100 pmol)
GST-
GtmA
b.
FeFsC (20 pmol)
-
IgM
(50 pmol)
GST-
GtmA

Supplementary Figure S2. Growth assays were established using *A. fumigatus* Δ*sidD* grown on MM without iron with discs supplemented with (i) FeFsC only, (ii) FeFsC mixed with anti-FeFsC IgM (IgM), or (iii) FeFsC mixed with GST-GtmA (negative control, at an equivalent protein concentration to the IgM). Plates were subsequently grown for **a.** 48 h, or **b.** 96 h. Anti-FeFsC IgM inhibits FeFsC-mediated growth restoration of *A. fumigatus* Δ*sidD* at a ratio of 50 pmol IgM/ 20 pmol FeFsC per disc after 96 h of growth (highlighted in red).

Acetylation of FeFsC with sulfo-NHS-acetate and generation of DAFC-NBD and uptake by A. fumigatus under iron starved conditions

RP-HPLC analysis revealed that a 2 molar excess of sulfo-NHS-acetate over FeFsC was the gave the highest percentage yield of FeDAFC, 46 %. A 4 molar and 8 molar excess gave a 35.5 % and 27 % yield respectively. This is due to the increase in concentration of the acetylating agent pushed the production of the triacetylated siderophore. RP-HPLC fluorescence detection analysis revealed a reduction in intensity of the signal of the DAFC-NBD reaction when compared to the NBD-X-SE control, 2261 and 6018 LU respectively (**Supplementary Figure 3a**). Further analysis by LC-MS confirmed the formation of FeDAFC-NBD by detection a singly charged ion corresponding to FeDAFC-NBD with an ammonium (NH_4) adduct (M: 1139.4, [M+H]+: observed *m/z* 1140.4; expected *m/z* 1140.4) (**Supplementary Figure 3b**). Fluorescence microscopy analysis was used to assess the uptake of FeDAFC-NBD by *A. fumigatus* mycelia.

Supplementary Table S1. Volumes of FeFsC, SNA and DMF required for the various reaction ratios.

Reaction Ratio	Volume FsC^{Fe3+} (µL)	Volume SNA (µL)	Volume DMF (µL)
1:2	373.5	14.25	42.75
1:4	373.5	28.50	28.5
1:8	373.5	57.00	-

a.

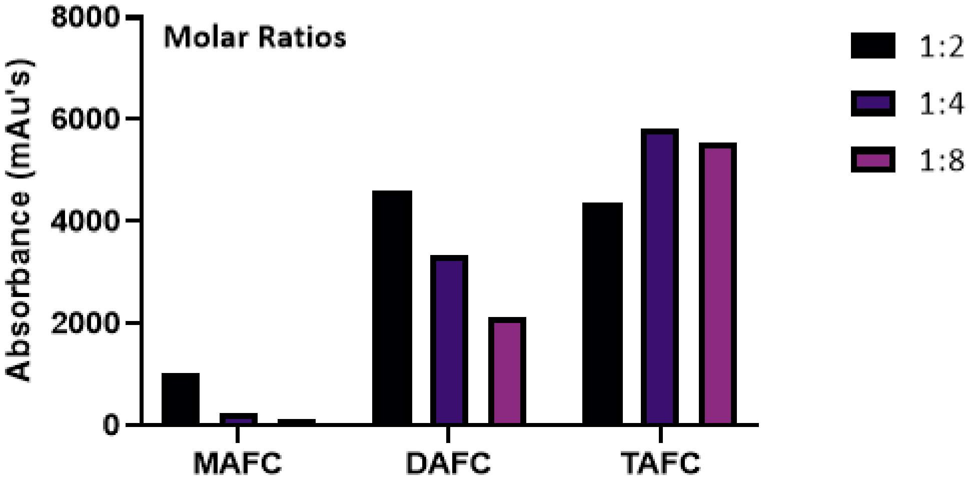
Molar Ratios
Absorbance (mAu's)
8000
6000
4000
2000
0
MAFC
DAFC
TAFC
1:2
1:4
1:8

b.

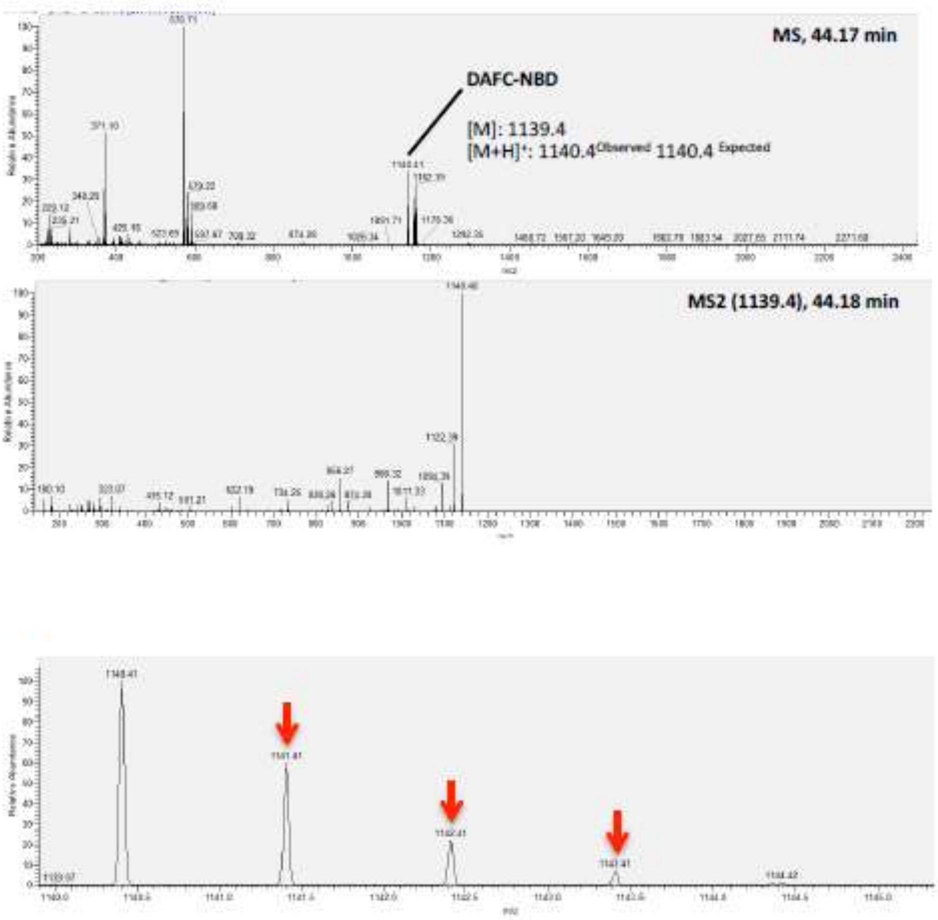
MS, 44.17 min
DAFC-NBD
[M]: 1139.4
[M+H]+: 1140.4 Observed 1140.4 Expected
MS2 (1139.4), 44.18 min

Supplementary Figure S3. Fluorescent FeDAFC, synthesised from FeFsC precursor, is acquired by *A. fumigatus* specifically under iron-deplete conditions. **a.** RP-HPLC analysis of the reaction of FeFsC with sulfo-NHS-acetate, with the intensity of the peaks for MAFC, DAFC and TAFC shown. The reaction was carried out with a 2, 4 and 8 molar excess of sulfo-NHS-acetate over FeFsC, in order to determine the optimum molar ratio to produce DAFC. **b.** LC-MS/MS identification of DAFC-NBD. DAFC-NBD reaction was analysed by LC-MS/MS. (A) MS (extracted ion chromatogram for *m/z* 1139 – 1142) spectrum shows the detection of DAFC-NBD as a singly charged ion (M: 1139.4, [M+H]+: observed *m/z* 1140.4; expected *m/z* 1140.4). MS2 spectrum shows the fragmentation of precursor ion. Increments of approx. +1.0 in observed *m/z* are indicative of C13 isotope incorporation in a singly charged ion.

REFERENCES

1. Moloney, N. Exploring the secreted proteome and metabolome of iron-starved As*pergillus fumigatus for* tools to combat fungal infection. PhD thesis. National University of Ireland, Maynooth, Maynooth, Ireland, **2017**. https://mural.maynoothuniversity.ie/id/eprint/16805/

2. Schrettl, M.; Bignell, E.; Kragl, C.; Sabiha, Y.; Loss, O.; Eisendle, M.; Wallner, A.; Arst, H.N., Jr.; Haynes, K.; Haas, H. Distinct roles for intra- and extracellular siderophores during Aspergillus fumigatus infection. *PLoS Pathog* **2007**, *3*, 1195-1207, doi:10.1371/journal.ppat.0030128.

3. Dolan, S.K.; Owens, R.A.; O'Keeffe, G.; Hammel, S.; Fitzpatrick, D.A.; Jones, G.W.; Doyle, S. Regulation of nonribosomal peptide synthesis: Bis-thiomethylation attenuates gliotoxin biosynthesis in Aspergillus fumigatus. *Chemistry and Biology* **2014**, *21*, 999-1012, doi:10.1016/j.chembiol.2014.07.006.
